# Angiotensin Converting Enzyme-2 (ACE-2) role in disease and future in research

**Published:** 2021-05-03

**Authors:** Amal Abdul-Hafez, Tarek Mohamed, Bruce D. Uhal

**Affiliations:** 1 Department of Pediatrics and Human Development, Michigan State University, USA; 2 Department of Physiology, Michigan State University, USA

**Keywords:** COVID-19, SARS-CoV-2, angiotensin converting enzyme-2 (ACE-2), renin angiotensin system, lung injury

## Abstract

Coronavirus Disease 2019 (COVID-19) is caused by the severe acute respiratory syndrome coronavirus 2 (SARS-CoV-2). Like the 2002–2003 epidemic severe acute respiratory syndrome coronavirus (SARS-CoV), angiotensin converting enzyme-2 (ACE-2) has been identified as the SARS-CoV-2 receptor.^1–3^ The virus docks into host cell via its spike protein binding to ACE-2 and undergoes proteolytic cleavage by TMPRSS2 protease to facilitate membrane fusion. The spike protein binding to ACE-2 has been shown to be stronger in the novel SARS-CoV-2 virus.^1^ This review will present an overview of ACE-2 biology.

## ACE-2 molecular/cellular biology

The human angiotensin converting enzyme 2 (ACE2) gene maps to the X chromosome, cytogenetic location Xp22.2. The full-length *ACE2* gene cDNA was cloned in 2000 by two independent research groups.^4,5^ EST database search for sequences showed its homology to the zinc metalloprotease angiotensin-I converting enzyme (ACE) which was also named angiotensin-converting enzyme homolog (ACEH).^4^ The ACE2 gene encodes a deduced 805-amino acid protein that shares approximately 40% identity with the N- and C-terminal domains of ACE. ACE2 contains a potential 17-amino acid N-terminal signal peptide and a putative 22-amino acid C-terminal membrane anchor. It has a conserved zinc metalloprotease consensus sequence (HEXXH) and a conserved glutamine residue that is predicted to serve as a third zinc ligand.^4,6^

The ACE2 enzyme is widely distributed on the human cells surface, especially the lungs. ACE2 receptors are also found in the heart, liver, digestive organs and kidneys and endothelial lining of vessels.^3,7^ Northern blot analysis detected high expression of ACE2 in kidney, testis, and heart, and moderate expression in colon, small intestine, and ovary.^4^

ACE2 mRNA was found to be expressed widely in human tissues and cells except red blood cells. Highest expression was detected in testis, renal and cardiovascular tissues, and in all portions of the gastrointestinal tract, particularly the ilium.^6,8^

The ACE2 gene contains 18 exons, with some similarity in exon size and organization to those of ACE, and spans approximately 40 kb of genomic DNA. The ACE2 gene contains an alternative splicing site for the 5′ untranslated exon 1 that was found to be expressed in the lung, testis, trachea, bronchial epithelial cells, small intestine, and various major organs.^4,9,10^ There are19 single nucleotide polymorphisms (SNPs) identified in the ACE2 gene, some of which have been associated with hypertension.^10,11^

**Figure 1:**
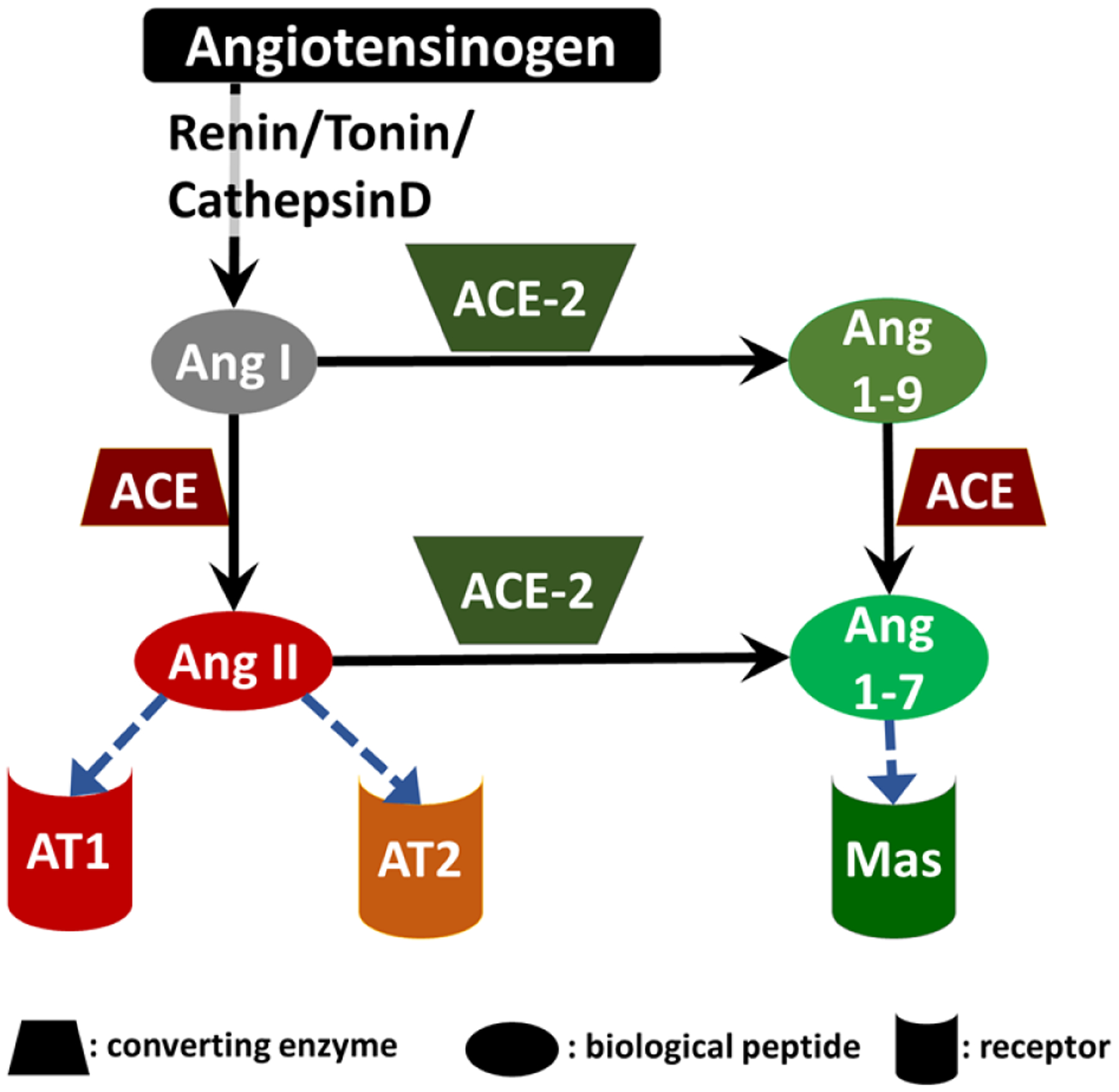
Schematic diagram of the Renin Angiotensin System (RAS).

The angiotensin converting enzyme 2 (ACE-2) was shown to play a protective role in the fibrogenesis and inflammation of many organs including liver and lung.^12,13^ ACE-2 is a part of the renin angiotensin system (Figure 1).

The renin angiotensin system (RAS) has been traditionally viewed as an endocrine system “Endocrine RAS” playing a significant role in blood pressure regulation. In the endocrine RAS, the kidney produced enzyme renin acts on circulating AGT protein. Renin cleaves AGT to produce a fragment of 10 amino acids known as angiotensin I (Ang I). Ang I is converted by angiotensin-converting enzyme (ACE) to the active octapeptide Ang II that exerts its actions through binding to specific cell surface angiotensin receptors. Two main receptors to Ang II have been identified; AT_1_ and AT_2_, both belong to superfamily of seven transmembrane G-protein coupled receptors. The AT_1_ receptor mediates all of the classical actions of Ang II (vasoconstriction, sodium retention, cell growth and proliferation), while AT_2_ receptor promotes vasodilation, cell differentiation, inhibition of cell growth and apoptosis and may play a counterbalancing role to the effects of Ang II on AT_1_ receptor.^14^ ACE-2 and its product angiotensin 1–7 (Ang 1–7) acting on mas oncogene receptor are referred to as “ACE-2/Ang1-7/Mas axis”, and have counteracting effects against the ACE/Ang II/AT_1_ axis of the RAS. Findings from numerous experimental studies have suggested notable protective effects of the ACE-2/Ang1-7/Mas axis in the cardiovascular system.^15^

ACE-2 is zinc-containing metalloenzyme and a membrane protein expressed in multiple organs such as heart, lungs, intestine, and kidneys.^5,16,17^ ACE-2 act as a counterbalance to the Angiotensin-converting enzyme (ACE), thus its decreased expression was found to associate with cardiovascular diseases.^18–20^ Full-length ACE2 consists of an N-terminal peptidase domain (PD) and a C-terminal collectrin-like domain (CLD) that ends with a single transmembrane helix and a ~40-residue intracellular segment.^5,21^ ACE2 cleaves Ang II to give Ang-(1–7) via the PD. ACE2 can also cleave Ang I to produce Ang-(1–9) which is then processed by other enzymes to become Ang-(1–7).^5,22,23^

## ACE-2 Enzyme function

The ACE-2 Enzyme is a glycosylated protein that functions exclusively as a carboxypeptidase cleaving angiotensin I (Ang I) and angiotensin II (Ang II), and is not inhibited by ACE inhibitors such as lisinopril.^4,24–26^ ACE2 is expressed predominantly in vascular endothelial cells of the heart and kidney. In addition to converting Ang II to Ang 1–7, ACE2 converts Ang I to Ang 1–9, which has 9 amino acids with no effect on blood vessels, but can be converted by ACE to Ang 1–7 that dilates blood vessels.^18,27^ ACE2 enzyme is important in the regulation of Ang II levels related to control of blood pressure and inflammation.^28^ In mice, deficiency of Ace2 was found to result in highly increased susceptibility to intestinal inflammation induced by epithelial damage via RAS-independent functions includinh; regulating intestinal amino acid homeostasis, expression of antimicrobial peptides, and effects on the gut microbiome.^29^

## ACE-2 animal models

Crackower et al. demonstrated that Ace2 maps to a defined quantitative trait locus (QTL) on the X chromosome in 3 different rat models of hypertension. In all hypertensive rat strains, Ace2 mRNA and protein expression were markedly reduced, suggesting that Ace2 is a candidate gene for this QTL. Targeted disruption of Ace2 in mice resulted in a severe cardiac contractility defect, increased AngII levels, and upregulation of hypoxia-induced genes in the heart. Genetic ablation of Ace on an Ace2 mutant background completely rescues the cardiac phenotype. This model showed that Ace2 is an essential regulator of heart function in vivo.^18^

Imai et al. reported that ACE2 and the AT_2_ receptor protect mice from severe acute lung injury induced by acid aspiration or sepsis.^7^ However, other components of the RAS, including ACE, Ang II, and the AT_1a_ receptor, promote disease pathogenesis, induce lung edemas, and impair lung function. Their study showed that mice deficient for ACE show markedly improved disease, and also that recombinant ACE2 can protect mice from severe acute lung injury thus identifying a critical function for ACE2 in acute lung injury.^7^

In a model for pulmonary fibrosis, Uhal et al. showed that ACE-2 mRNA and activity were decreased in the lungs of bleomycin-treated rats and C57-BL6 mice similar to ACE-2 decrease in pulmonary fibrosis patients.^12^ In mice exposed to low doses of bleomycin, lung collagen accumulation was enhanced by intratracheal administration of either ACE-2-specific small interfering RNAs (siRNAs) or the peptide DX(600), a competitive inhibitor of ACE-2. Administration of either ACE-2 siRNA or DX(600) significantly increased the Ang II content of mouse lung tissue above the level induced by bleomycin alone. Coadministration of the Ang II receptor antagonist saralasin blocked the DX(600)-induced increase in lung collagen. Moreover, purified recombinant human ACE-2, delivered to mice systemically by osmotic minipump, attenuated bleomycin-induced lung collagen accumulation. This study suggest that ACE-2 protects against lung fibrogenesis by limiting the local accumulation of the profibrotic peptide Ang II.^12^

Gurley et al. generated Ace2-deficient mice and found that they were viable, fertile, and had normal cardiac dimensions and function.^30^ After acute Ang II infusion, plasma concentrations of Ang II increased almost 3-fold higher in Ace2-deficient mice than in controls. In a model of Ang II-dependent hypertension, blood pressures were substantially higher in the Ace2-deficient mice than in wildtype mice, and severe hypertension in Ace2-deficient mice was associated with exaggerated accumulation of Ang II in the kidney. Although absence of functional ACE2 caused enhanced susceptibility to Ang II-induced hypertension, the authors found no evidence for a role of ACE2 in the regulation of cardiac structure or function. This model suggested that ACE2 is a functional component of the renin-angiotensin system, metabolizing Ang II and thereby contributing to the regulation of blood pressure.^30^

McCray et al.^31,32^ produced the K18-hACE2 transgenic mouse for coronavirus research, currently being introduced by the Jackson laboratories for use in COVID-19 research. In this model, the human cytokeratin 18 (K18) promoter regulates human ACE2 gene expression in epithelial cells. The K18-hACE2 transgenic mouse will exhibit fatal infection when infected with a human SARS-CoV strain via intranasal inoculation. The infection would spread in the mouse model lungs, affecting airway epithelium and the alveoli with subsequent spread to the brain. The infection causes both lungs and brain inflammation, characterized by up-regulation of pro-inflammatory cytokines and chemokines. In addition, the lungs exhibit infiltration of macrophages and lymphocytes. The transgene expression of hACE2 in epithelial cells caused fatal SARS-CoV infection in the K18-hACE2 mice; showing symptoms of weight loss, lethargy, labored breathing, and death.^32^

## ACE-2 roles in disease and therapy

### ACE-2 and Influenza Infections:

ACE2 was shown to be associated with the acute lung injury caused by influenza virus.^33^ In the lung, ACE2 is found primarily in epithelial cells,^34^ and the ACE2/angiotensin-(1–7)/Mas axis directly regulates epithelial cell survival.^35,36^ In mice, ACE2 is a mediator of the acute lung injury caused by influenza A H5N1- and H7N9-virus infection,^37,38^ and in patients, increased ACE2 levels are associated with severe disease.^37,39^ In mice experimentally infected with H5N1 influenza, treatment with an ARB (losartan) improves survival.^33,40^

In an experimental mouse model, Yang et al. showed that ACE2 mediates avian-origin influenza A (H7N9) virus-induced acute lung injury and that ACE2 deficiency worsened the disease pathogenesis markedly by targeting the AT_1_ receptor.^38^ In H7N9-infected patients, Huang et al.^39^ showed that plasma levels of Ang II are markedly elevated and are associated with disease progression. Moreover, the sustained high levels of Ang II in these patients are strongly correlated with mortality. These findings indicate that angiotensin II is a biomarker for lethality in flu infections.^39^

In experimental mouse models of infection with highly pathogenic avian influenza A H5N1 virus, Zou et al.^37^ showed downregulation of angiotensin-converting enzyme 2 (ACE2) expression in the lung and increased serum angiotensin II levels. Genetic inactivation of ACE2 caused severe lung injury in H5N1-challenged mice, confirming a role of ACE2 in H5N1-induced lung pathologies. Administration of recombinant human ACE2 ameliorated avian influenza H5N1 virus-induced lung injury in mice. These data link H5N1 virus-induced acute lung failure to ACE2 and provide a potential treatment strategy to address future flu pandemics.^37^

### Angiotensin Converting Enzyme-2 (ACE-2) in tissue injury and fibrosis:

ACE-2 and its product Ang 1–7 were shown to play a protective role in experimental models of fibrosis. In cardiac fibrosis, ACE-2 expression was shown to be protective against Ang II-induced and hypertension-induced cardiac fibrosis.^41^ In liver fibrosis, both Ang 1–7 and ACE-2 provide protection against the development of liver injury and progression to cirrhosis.^42^ In experimental acute lung injury, ACE-2 also has protective effect associated with reduced levels of Ang II. Earlier studies of knockout mice have shown a clear protective effect of ACE-2 on experimental acute lung injury in response to acid aspiration or sepsis; the protective effect of ACE-2 was associated with reduced levels of Ang II after experimental lung injury.^24^ A study on rat brain astrocytes showed that Ang II down-regulates ACE-2 mRNA through angiotensin receptor I in a positive feed-forward system that favors Ang II – mediated responses.^43^ In the lung models of fibrosis, ACE-2 was shown to regulate alveolar epithelial cell survival by balancing the proapoptotic Ang II and its antiapoptotic degradation product Ang1-7 through its receptor “mas”.^12,36^

### ACE-2 and liver fibrosis:

The role of ACE-2 in liver disease is of special interest as several lines of evidence suggest that the RAS also participates in the regulation of hepatic inflammation, tissue remodeling, and fibrosis after liver injury analogous to other organs. RAS induces key steps involved in hepatic fibrosis, such as activation of hepatic stellate cells and expression of transforming growth factor β1.^44^ Treatment with angiotensin-converting enzyme inhibitors, and angiotensin receptor blockers attenuate fibrosis progression in both animal and human studies^45^. Additionally, supplementation of ACE-2 can prevent liver fibrosis of bile duct ligation mouse model.^13,45^

## ACE-2 in nephropathies

Low ACE2 levels have been reported in established renal disease and in the 5/6ths nephrectomy model of renal insufficiency. There is evidence that acquired or genetically determined ACE2 deficiency may enhance histologic damage and increase proteinuria in experimental nephropathies.^46^

Substantial experimental data suggest that ACE2 is protective in diabetic nephropathy. Numerous studies have demonstrated an attenuation of ACE2 expression in the glomeruli in diabetes models.^47–50^ associated with an increase in ACE expression in the glomeruli and in the vasculature.^51^ Studies in human showed that ACE2 was decreased and ACE expression increased in both the tubulointerstitium and glomeruli in patients with diabetes.^52^

## ACE-2 and Pulmonary Fibrosis

ACE-2 has been shown to play an established protective role in lung disease through effects mediated by Mas oncogene, the ACE-2 peptide product ANG1–7 receptor^12,53–55^. Previous studies from our lab and other groups suggest that ACE-2 is down-regulated in fibrotic conditions of the adult and neonatal human lung^12,53,54^ via Mas receptor mechanisms^36^. Data from our lab was first to discover the significant decrease of ACE-2 in the human IPF lung and identify the protective effects of ACE-2 in the IPF disease^12^. We also demonstrated that ACE-2 regulates alveolar epithelial cell survival by balancing the proapoptotic Ang II and its antiapoptotic degradation product Ang 1–7 through the Ang 1–7 action on its receptor “mas”.^36^

The alveolar epithelial type II cells are considered lung alveolar “stem cells”.^56,57^ They represent a major source of ACE-2 in the adult lung, are normally quiescent but actively proliferate in lung fibrosis due to lung injury and downregulate this protective enzyme. In our studies we found that in lung biopsy specimens obtained from IPF patients, immunoreactive ACE-2 was absent in alveolar epithelia that were positive for proliferation markers but was robustly expressed in alveolar epithelia devoid of proliferation markers. This explained the loss of ACE-2 in lung fibrosis and demonstrated cell cycle-dependent regulation of this protective enzyme.^58^

## ACE-2 and Bronchopulmonary Dysplasia

Bronchopulmonary dysplasia (BPD) is recognized as a chronic lung disease of infancy that presents as a systemic syndrome and can be associated with neurodevelopmental deficits, cognitive impairments, failure to thrive, pulmonary hypertension and cor pulmonale.^59^ Supplemental oxygen, which is frequently used in the treatment of pulmonary insufficiency in premature infants, has been implicated in the development of BPD.^53,54^ In adult animal models of acute lung injury,^7,60^ ACE-2 was shown to inhibit lung edema formation and inflammation as well as fibrogenesis. However, little is known about the role of ACE-2 in neonateal models of BPD. Our research group showed that ACE-2 is expressed in fetal human lung fibroblasts but is significantly decreased by hyperoxic gas lung injury,^54^ an effect reversed when hyperoxia preceded by hypoxia.^53^ Furthermore, Wagenaar *et al.* showed that Mas receptor agonists reduce inflammation of the oxygen-induced lung injury in rats.^55^

Chorioamnionitis and mechanical ventilation are also associated with bronchopulmonary dysplasia (BPD) in preterm infants.^61,62^ A study by Hillman *et. al.*, on neonatal lamb model of chorioamnionitis and infection showed altered ratio of ACE-1 to ACE-2.^62^ Although in adults, a recent pilot clinical trial of a recombinant form of human angiotensin-converting enzyme 2 (rhACE-2) was performed in adults with acute respiratory distress syndrome. As a result of treatment, surfactant protein D concentrations were increased and there was a trend for a decrease in interleukin-6 concentrations in rhACE-2-treated subjects compared with placebo.^63^ This shows potential use of ACE2 as a therapeutic for neonates as well.

## Role of ACE2 in coronavirus infection

The RAS is involved in lung injury, cardiovascular functions, and coronavirus infections.^1,3,18,25–28,64–67^ COVID-19 binds to a specific ACE2 receptor that is located in the lungs within bronchioles and alveoli and other tissues in the body, including those of the kidney and small intestine.^36,68–70^ The majority of these ACE2 enzymes are fixed to cell surfaces, mainly on the endothelium.^71^

Coronavirus that causes severe acute respiratory syndrome (SARS) utilize their Spike (S) to associate with cellular receptors for target cells entry. When isolated from SARS coronavirus-permissive cells, ACE-2 was found to efficiently bind the S1 domain of the SARS-CoV-1 S protein and proved to be a functional receptor for SARS-CoV.^6,72^ The SARS-CoV-1 S protein was also found to exaggerate acute lung failure through deregulation of the RAS, while blockage of AT_1_ receptor, which mediates Ang II-induced vascular permeability and severe acute lung injury, attenuated S protein-induced lung injury in mice.^73^ Another novel group of human coronaviruses called NL63 was discovered in patients with respiratory tract illness, and was found to also employ ACE-2 as a receptor to mediate infection.^74^

Severe acute respiratory syndrome coronavirus-2 (SARS-CoV-2) is a novel coronavirus that has caused a worldwide pandemic of the human respiratory illness COVID-19, resulting in a severe threat to public health and safety.^75^ Like the SARS virus CoV-1, the CoV-2 virus enters cells by attachment to ACE-2 receptors while the viral S protein is processed (or primed) by the cellular protease TMPRSS2.^1–3,76^ However, the spike protein binding to ACE-2 has been shown to be stronger in the novel SARS-CoV-2 virus.^1^ The S1 C-terminal domain (CTD) has been identified as the key region of SARS-CoV-2 involved in interaction with human ACE2.^77^

Yan et al. presented cryoelectron microscopy structures of full-length human ACE2 with or without the receptor-binding domain of the surface spike glycoprotein of SARS-CoV-2. The receptor-binding domain is recognized by the extracellular peptidase domain of ACE2 mainly through polar residues.^23^ Wang et al. determined the 2.5-angstrom crystal structure of SARS-CoV-2 CTD in complex with human ACE2 and found that the receptor-binding mode was similar to that of SARS-CoV-1, but that SARS-CoV-2 had slightly stronger affinity due to key substitutions in the binding interface. Antibodies against the SARS-CoV-1 receptor-binding domain did not interact with the SARS-CoV-2 S protein, confirming important structural differences between the 2 viruses.^6,77^

As SARS-COV-2 binds the ACE-2 entry receptor, direct viral cellular damage, release of excessive immune mediators and viral particles in the lung tissue, and coagulation abnormalities occur.^69,78,79^ Elevated levels of immune factors released, such as interleukins, tumor necrosis factor and interferons contribute to trigger the associated cytokine storm in COVID-19.^80,81^ Immune-related mechanisms are thought to be responsible for the disseminated intravascular coagulation with lung micro-thrombosis in COVID-19 pneumonia.^82,83^ In severely affected COVID-19 patients, renal dysfunction is thought to be due to the presence of ACE-2 receptors in the kidney.^84,85^

## Future in ACE-2 research

For its protective role in several organs, ACE-2 activation or supplementation is being considered as a therapeutic approach.^25,26,86–89^

Hernández Prada et al. identified compounds that enhance ACE2 activity using conformation-based rational drug discovery strategy and to evaluate whether such compounds reverse hypertension-induced pathophysiology.^88^ In vitro assays revealed 2 compounds (a xanthenone and resorcinolnaphthalein) that enhanced ACE2 activity in a dose-dependent manner. Acute in vivo administration of the xanthenone resulted in a dose-dependent transient and robust decrease in blood pressure of spontaneously hypertensive rats. Chronic infusion of the xanthenone resulted in a modest decrease in the spontaneously hypertensive rat blood pressure, whereas it had no effect in Wistar-Kyoto rats. The decrease in blood pressure was also associated with improvements in cardiac function and reversal of myocardial, perivascular, and renal fibrosis in the spontaneously hypertensive rats. They concluded that activating ACE2 can help decrease blood pressure for future antihypertensive therapy.^88^

Agents that stimulate Mas, the end receptor of the ACE-2 product Ang1-7, have been studied in preclinical studies, especially for treatment of hypertension. The orally active nonpeptide drug, AVE 0991, is a AT2R/Mas agonist. AVE 0991 binding to aortic endothelial cell membranes, induces vasorelaxation in rats and acts through a Mas-mediated mechanism.^90–92^ Novel peptide Mas agonist drugs CGEN-856S and CGEN-857 induce vasorelaxation in murine aortic rings and a dose-dependent decrease in mean arterial pressure in spontaneously hypertensive rats.^93^ Since Ang1-7 has a short half-life and is easily degraded, a few studies aimed at using Ang1-7 analogues that are more stable. HPβCD- Ang-(1–7) is a stable Ang-(1–7) analogue. The hydroxypropyl-β-cyclodextrin protects Ang-(1–7) from digestive tract enzymes. Chronic oral administration lowers BP in rats following ischemia-reperfusion injury.^94^ Cyclic Ang-(1–7) cAng1-7 is another peptidase resistant Ang-(1–7) analogue. It improved endothelial function post-MI in male Sprague Dawley rats as well as improved peripheral endothelium-dependent vasodilation, as measured in isolated aortic rings.^95^

A few studies have utilized ACE-2-primed endothelial progenitor cells (ACE2-EPCs) to induce protective effects on endothelial cells through their released exosomes.^96–98^ ACE2 overexpression can enhance the protective effects of EPCs on endothelial cells injury, majorly through the exosomal effects on mitochondrial function and down-regulating the Nox2/ROS pathway.^96–98^

Recombinant human ACE-2 has been tested in healthy individuals in clinical trials to determine medication pharmacokinetics and pharmacodynamics^99^, and has been investigated as pipeline drug in a pilot clinical trial to treat adult acute lung injury^63^. ACE-2 targeted therapies might be future beneficial treatments for adult and neonatal lung disease.^26,100^

ACE2 has become the focus of COVID-19 research and drug development efforts. Among the novel compounds under development is human recombinant soluble ACE2 (hrsACE2 [APN01; Apeiron Biologics, Vienna, Austria]).^101^ A recent study by Zoufaly et al. published in The Lancet Respiratory Medicine describes encouraging data from the first severe COVID-19 patient successfully treated with human recombinant soluble angiotensin-converting enzyme-2 (hrsACE2).^102^ The rationale for their study was based on two mechanisms of action that theoretically should be of benefit in COVID-19.^103^ The first involves binding the viral spike protein and thereby neutralizing SARS-CoV-2,^104^ and the second is minimizing injury to multiple organs, including the lungs, kidneys, and heart, because of unabated renin–angiotensin system hyperactivation and increased angiotensin II concentrations.^18,49,73^ The hrsACE2 was previously tested in 89 patients, namely in healthy volunteers in phase 1 studies and in patients with acute respiratory distress syndrome (ARDS) in phase 2 clinical studies, with an acceptable safety profile.^63,99^ Moreover, hrsACE2 can reduce SARS-CoV-2 load by a factor of 1000–5000 in in-vitro cell-culture experiments and engineered organoids, directly demonstrating that ACE2 can effectively neutralize SARS-CoV-2.^104^ Data from Zoufaly et al. document upon treatment of an adaptive immune response, the disappearance of the virus swiftly from the serum, the nasal cavity and lungs, and a reduction of inflammatory cytokine levels that are critical for COVID-19 pathology. Notably, the use of hrsACE2 did not impede the generation of neutralizing antibodies, leading to a significant clinical improvement of the treated patient.^102,105^

## Summary and Conclusions

ACE-2 is a protective enzyme to many organs that locally express components of the renin angiotensin system (RAS) within its tissues. The protective mechanisms of the ACE-2 enzyme are attributed to its role in degrading the pro-injury/pro-fibrotic peptide AngII and producing the protective/antiapoptotic peptide Ang1-7 that acts on Mas receptor in what’s referred to as “ACE-2/Ang1-7/Mas axis” or “the protective arm of the RAS”. Agonists of this protective arm are suggested as therapy for the multiple diseases in which RAS is involved. Most recently, as the SARS-Cov-2 receptor, ACE-2 has been the focus of research due to the COVID-19 pandemic. This lead researchers to revisit the use of recombinant human ACE-2 as a future therapy for lung diseases.
